# Correction: PIWIL2 restrains the progression of thyroid cancer via interaction with miR-146a-3p

**DOI:** 10.1186/s12902-024-01786-z

**Published:** 2024-11-26

**Authors:** Xiaoxiao Lu, Qingyun Zhu, Hong Du, Mingjun Gu, Xiangqi Li

**Affiliations:** 1 Department of Endocrinology and Metabolism, Punan Hospital, Pudong New Area, Shanghai, 200125 China; 2 Department of Intervention, Gongli Hospital, Naval Medical University, Shanghai, 200135 China; 3 Department of General Practice, Hudong Community Health Service Centre, Pudong New Area, Shanghai, 200129 China; 4 Department of Endocrinology and Metabolism, Gongli Hospital, Naval Medical University, Shanghai, China

**Correction: BMC Endocr Disord 23, 184 (2023)**

10.1186/s12902-023-01416-0

Following publication of the original article [1], the authors reported mistakes in Figs. 6 and 7, and requested to update the figures.

The original published Fig. 6 was:

**Fig. 6:**
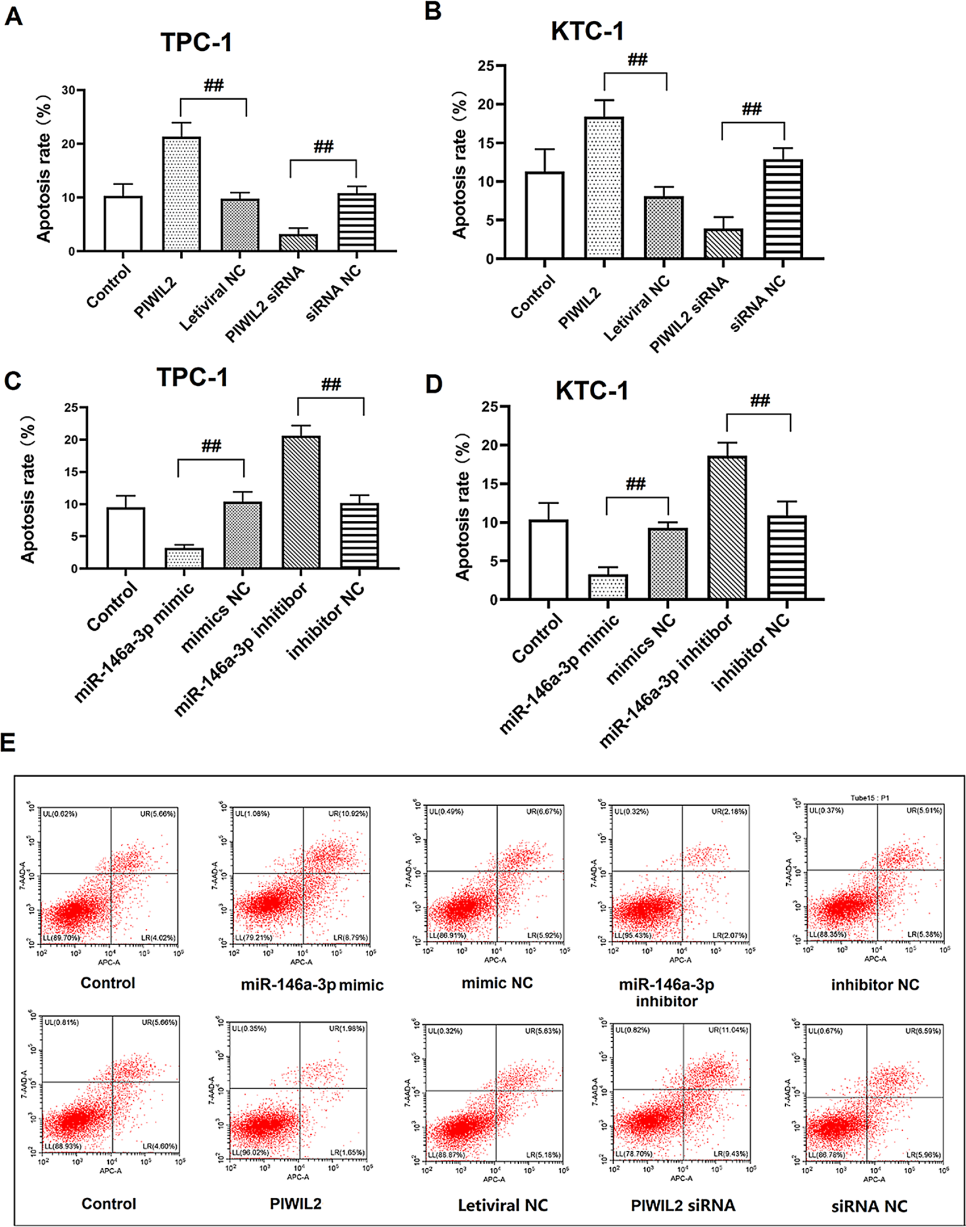
PIWIL2 and miR-146a-3p regulated the apoptosis of TC cell lines. **(A)** The apoptosis of TPC-1 cells after over-expressed miR-146a-3p or knockdown miR-146a-3p. **(B)** The apoptosis of TPC-1 cells after over-expressed PIWIL2 or knockdown PIWIL2. **(C)** The apoptosis of KTC-3 cells after over-expressed miR-146a-3p or knockdown miR-146a-3p. **(D)** The apoptosis of KTC-3 cells after over-expressed PIWIL2 or knockdown PIWIL2. **(E)** The flow cytometry analysis of apoptosis of TPC-1 cells after over-expressed miR-146a-3p, knockdown miR-146a-3p, over-expressed PIWIL2 or knockdown PIWIL2. ^##^: *P* < 0.01 between groups. *N* = 6. TC: thyroid cancer

The correct Fig. 6 should read:

**Fig. 6:**
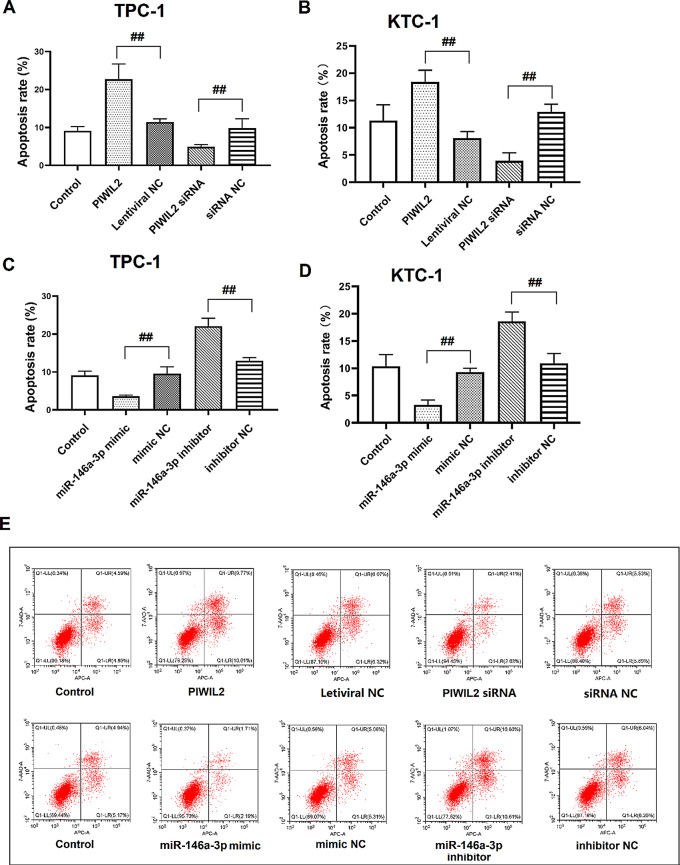
PIWIL2 and miR-146a-3p regulated the apoptosis of TC cell lines. **(A)** The apoptosis of TPC-1 cells after over-expressed miR-146a-3p or knockdown miR-146a-3p. **(B)** The apoptosis of TPC-1 cells after over-expressed PIWIL2 or knockdown PIWIL2. **(C)** The apoptosis of KTC-3 cells after over-expressed miR-146a-3p or knockdown miR-146a-3p. **(D)** The apoptosis of KTC-3 cells after over-expressed PIWIL2 or knockdown PIWIL2. **(E)** The flow cytometry analysis of apoptosis of TPC-1 cells after over-expressed miR-146a-3p, knockdown miR-146a-3p, over-expressed PIWIL2 or knockdown PIWIL2. ^##^: *P* < 0.01 between groups. *N* = 6. TC: thyroid cancer

The original published Fig. 7 was:

**Fig. 7:**
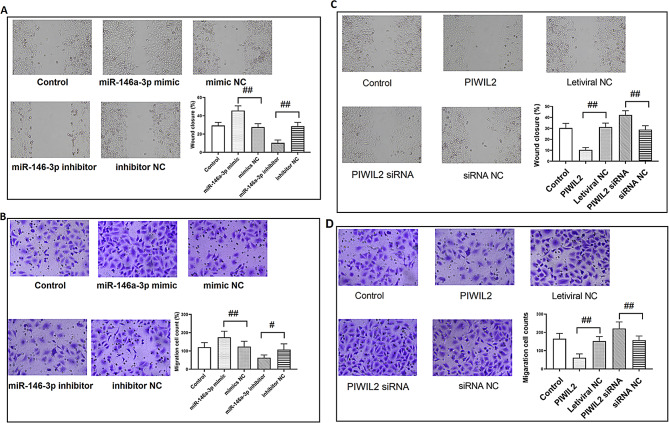
miR-146a-3p and PIWIL2 regulated the migration and invasion of TC cell lines. **(A)** The migration of TPC-1 cells after miR-146a-3p was overexpressed or knocked down. **(B)** The invasion of TPC-1 cells after miR-146a-3p was overexpressed or knocked down. **(C)** The migration of TPC-1 cells after PIWIL2 was overexpressed or knocked down. **(D)** The invasion of TPC-1 cells after PIWIL2 was overexpressed or knocked down. ^#^: *P* < 0.05 between groups; ^##^: *P* < 0.01 between groups. *N* = 6. TC: thyroid cancer

The correct Fig. 7 should read:

**Fig. 7:**
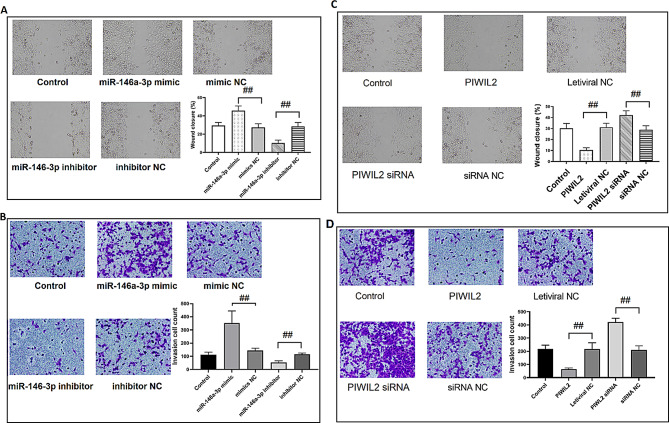
miR-146a-3p and PIWIL2 regulated the migration and invasion of TC cell lines. **(A)** The migration of TPC-1 cells after miR-146a-3p was overexpressed or knocked down. **(B)** The invasion of TPC-1 cells after miR-146a-3p was overexpressed or knocked down. **(C)** The migration of TPC-1 cells after PIWIL2 was overexpressed or knocked down. **(D)** The invasion of TPC-1 cells after PIWIL2 was overexpressed or knocked down. ^#^: *P* < 0.05 between groups; ^##^: *P* < 0.01 between groups. *N* = 6. TC: thyroid cancer

The original article [1] has been updated.

## References

[1] Lu X, Zhu Q, Du H, et al. PIWIL2 restrains the progression of thyroid cancer via interaction with miR-146a-3p. BMC Endocr Disord. 2023;23:184. 10.1186/s12902-023-01416-0. 37641092 10.1186/s12902-023-01416-0 PMC10464277

